# Alaska pollock protein as a functional dietary source for promoting skeletal muscle hypertrophy and lipid metabolic remodeling

**DOI:** 10.1371/journal.pone.0348366

**Published:** 2026-05-13

**Authors:** Ayumu Kojima, Risa Mukai, Mizuki Morisasa, Wakako Tawara, Shunsuke Amagaya, Kenjiro Furusho, Norika Tsutsumi, Yukina Tadokoro, Haruto Otsuka, Miu Fujii, Taro Kishida, Atsushi Kuno, Azusa Tomioka, Shinji Okada, Tsukasa Mori, Naoko Goto-Inoue

**Affiliations:** 1 Department of Marine Science and Resources, College of Bioresource Sciences, Nihon University, Fujisawa, Kanagawa, Japan; 2 Graduate School of Agriculture, Ehime University, Matsuyama, Japan; 3 Molecular and Cellular Glycoproteomics Research Group, Cellular and Molecular Biotechnology Research Institute, National Institute of Advanced Industrial Science and Technology, Ibaraki, Japan; 4 Department of Food and Life Sciences, Faculty of Food and Nutritional Sciences, Toyo University, Asaka, Saitama, Japan; Fujita Health University, JAPAN

## Abstract

Alaska pollock protein (APP) promotes skeletal muscle hypertrophy, particularly in fast-twitch fibers. Using a time-controlled feeding model, plasma collected 90 min after APP intake markedly enhanced C2C12 myotube hypertrophy and contractility. At this time point, plasma glucagon-like peptide-1 levels were significantly elevated, suggesting the involvement of circulating factors associated with APP intake. Proteomic and lipidomic analyses of skeletal muscle identified 2302 proteins and 538 lipids, and revealed pronounced remodeling of lipid metabolism, including a 127% increase in docosahexaenoic acid (DHA)-containing phospholipids in hypertrophied fast-twitch fibers, partially resembling endurance training adaptations. APP also upregulated adipose triglyceride lipase and hormone-sensitive lipase in the skeletal muscle, indicating enhanced lipid catabolism. Although APP contained only trace amounts of DHA (~0.008% of the diet), this level was substantially lower than the dietary DHA level previously reported to alter tissue lipid composition. Therefore, APP-derived DHA was unlikely to be the primary driver of the observed phenotype. Together, these findings indicate that APP functions primarily as a high-quality protein source that promotes muscle anabolic responses and is associated with remodeling of lipid metabolic pathways, thereby potentially contributing to a circulating environment favorable for skeletal muscle hypertrophy and metabolic regulation.

## Introduction

Skeletal muscle serves as the primary organ for locomotion and is instrumental in whole-body metabolism by sustaining basal energy expenditure, regulating glucose and lipid homeostasis, supporting thermogenesis, and acting as an endocrine organ through myokine secretion, thereby exerting diverse physiological effects [1,2]. Consequently, maintaining skeletal muscle mass and function is essential for preventing metabolic disorders and conditions, such as sarcopenia, thereby preserving overall health and quality of life. Adequate nutritional intake and exercise are indispensable for preserving and increasing muscle mass, with dietary proteins being particularly critical. Proteins provide the amino acids required for muscle protein synthesis and activate intracellular signaling pathways that promote hypertrophy [3–5]. The anabolic effects of dietary proteins have traditionally been attributed to essential amino acids, particularly branched-chain amino acids, such as leucine, which stimulates muscle protein synthesis by activating the mechanistic target of rapamycin (mTOR) signaling pathway [4]. However, recent studies suggest that besides amino acid composition, food-derived bioactive peptides and other components influence muscle protein metabolism [6].

Considering the critical role of dietary proteins in supporting muscle mass and function, attention has increasingly turned to identifying high-quality protein sources with superior bioavailability and anabolic potential. Fish protein represents one such source, offering excellent biological value and considerable global dietary importance. Among fish species, Alaska pollock (*Theragra chalcogramma*) is widely consumed in Japan, particularly in processed seafood products such as surimi, fish sausages, and imitation crab. Alaska pollock protein (APP) is characterized by its low lipid and carbohydrate content and high protein concentration. Using the indicator amino acid oxidation method, APP achieved a utilization efficiency score (104) exceeding that of egg proteins (100), and nitrogen balance analyses further confirmed its remarkably high amino acid bioavailability, underscoring APP as a superior dietary protein source [7]. In our previous study, short-term APP intake (7–14 d) induced significant hypertrophy of the gastrocnemius muscle in rats, characterized by a marked enlargement of fast-twitch fibers [7,8]. Activation of the Akt–mTOR signaling pathway, a central regulator of muscle protein synthesis, suggests its involvement in APP-induced hypertrophy [8]. However, APP-induced hypertrophy may instead arise from suppressed proteolytic pathways rather than enhanced protein synthesis [7]. The precise molecular mechanisms remain unclear, in part due to limitations of earlier experimental designs. Previous studies have suggested that APP-derived protein components exert metabolic effects that cannot be explained solely by amino acid composition, indicating a potential role of bioactive peptides in regulating systemic metabolism [9], including glucose and lipid metabolism. Most studies employed long-term *ad libitum* feeding models [7,10], wherein variability in feeding timing may obscure rhythmic changes in muscle protein synthesis and degradation, thereby complicating the interpretation of upstream signaling and metabolic adaptations.

To address these limitations and clarify the molecular mechanisms underlying APP-induced muscle hypertrophy, the present study established a novel experimental model with strict control of feeding timing. Time-dependent changes following APP ingestion were investigated by synchronizing oral administration at the end of a 14-d *ad libitum* feeding period, with plasma and skeletal muscle samples collected at 90 and 210 min postprandially for analysis of protein and lipid content. To further explore the effects of plasma-derived factors on skeletal muscle, *in vitro* experiments using cultured myotubes were performed. Proteomic analyses indicated alterations in lipid metabolic pathways in skeletal muscle, prompting comprehensive lipidomic analyses of plasma, skeletal muscle, and liver to characterize metabolic adaptations associated with APP intake. This study provides new insights into the mechanisms by which APP promotes skeletal muscle hypertrophy and highlights the role of diet-derived factors in regulating muscle growth.

## Materials and methods

### Animal model

Five-week-old male Sprague–Dawley rats (SLC, Shizuoka, Japan) were housed in stainless steel wire-mesh cages in a temperature-controlled room (24 ± 1°C) under a 12-h light–dark cycle (dark phase: 15:00–03:00). After a 5-d acclimation period, the rats were randomly assigned to one of two dietary groups: (1) the casein diet group, which received the AIN-93 diet containing casein as the protein source, or (2) the APP diet group, in which casein was replaced with APP. The diets were formulated to be isonitrogenous, and the total nitrogen content was matched between groups [7]. Both diets were provided *ad libitum* for 14 d. On the final day, rats were fasted for 3 h prior to the onset of the dark phase and subsequently received a single oral dose equivalent to one-sixth of their daily feed allocation, corresponding to the estimated amount consumed during a single spontaneous meal [11]. Animals were randomly assigned to each experimental group (n = 8 per group). Animals were euthanized by decapitation 90 or 210 min after administration. Trunk blood was immediately collected from the carotid artery into tubes containing EDTA and a dipeptidyl peptidase 4 (DPP-4) inhibitor to prevent glucagon-like peptide-1 (GLP-1) degradation. Plasma was separated by centrifugation and stored at −80°C until analysis. Gastrocnemius muscle samples were collected at the corresponding time points. Liver and serum samples were collected after 2 weeks of feeding. All animal procedures were reviewed and approved by the Animal Care and Use Committee of Ehime University (Approval No. 08A92 (2023)). All experimental protocols were conducted in accordance with the institutional guidelines for animal care and use. Animals were euthanized using isoflurane, in accordance with approved protocols.

### Cell culture

C2C12 myoblasts (American Type Culture Collection, Manassas, VA, USA) were seeded in 12-well plates (1 mL/well) or 48-well plates (200 μL/well) in Dulbecco’s modified Eagle’s medium (DMEM; 25 mM glucose; Thermo Fisher Scientific, Waltham, MA, USA) supplemented with 10% fetal bovine serum (BioWest, Nuaillé, France) and 1% penicillin–streptomycin. Cells were maintained at 37°C in a humidified incubator with 5% CO₂. When the cells reached approximately 80% confluence, the medium was replaced with differentiation medium consisting of DMEM supplemented with 2% horse serum (Serana Europe GmbH, Brandenburg, Germany), 1% nonessential amino acids (Thermo Fisher Scientific), and 1% penicillin–streptomycin.

For the 12-well plate experiments, after 4 days of differentiation, the medium was replaced with differentiation medium containing 0.5% rat plasma collected from either casein-fed or APP-fed rats at 90 or 210 min post-feeding. Rat plasma was added at a final concentration of 0.5%, a level selected to maintain cell viability while allowing detection of physiologically relevant circulating factor–mediated effects. Cells were incubated under these conditions for 48 h. For the 48-well plate experiments, the medium was similarly replaced with differentiation medium containing 0.5% rat plasma collected from the same experimental groups and time points, and cells were maintained for 72 h. The duration of plasma treatment was adjusted according to the experimental endpoint, while all evaluations were performed on day 6 of differentiation. For contractility measurements, plasma treatment was initiated on day 4 of differentiation and continued for 48 h until day 6. For fusion index measurements, plasma treatment was initiated on day 3 of differentiation and continued for 72 h until day 6.

### Immunofluorescence staining and fusion index analysis

Myotubes were fixed with 4% paraformaldehyde and washed twice with phosphate-buffered saline containing Tween 20 (PBST). The cells were then blocked with Blocking One Histo (Nacalai Tesque, Tokyo, Japan) for 20 min, and were incubated overnight at 4°C with primary antibodies against myosin heavy chain (MF20, Development Studies Hybridoma Bank, University of Iowa, IA, USA). Plates were subsequently washed three times with PBST and incubated for 2 h at 22°C–25°C with Alexa Fluor 488-conjugated secondary antibodies (#A11029; Thermo Fisher Scientific). Following three additional PBST washes, cells were incubated with 4′,6-diamidino-2-phenylindole, dihydrochloride (Thermo Fisher Scientific) for 15 min to stain nuclei. Fluorescence images were acquired using a wide-field fluorescence microscope (BZ-X810; Keyence, Osaka, Japan). The proportion of positive areas and number of nuclei per myotube were quantified to calculate the fusion index.

### Muscle histology and fiber type analysis

Frozen gastrocnemius muscles were sectioned at a thickness of 10 μm using a cryostat (CM1950; Leica Microsystems, Wetzlar, Germany) and mounted onto adhesive-coated slides (Matsunami, Osaka, Japan). To identify muscle fiber types, sections were subjected to immunofluorescence staining using fiber type-specific antibodies. Sections were fixed with acetone and incubated overnight at 4°C with primary antibodies against MyHC IIa (SC-71) and MyHC IIb (BF-F3), obtained from the Developmental Studies Hybridoma Bank (University of Iowa, Iowa City, IA, USA). Following five washes with PBST, sections were incubated for 1 h at 22°C–25°C with secondary antibodies conjugated to Alexa Fluor 488 (A21042) or Alexa Fluor 555 (A21127; Thermo Fisher Scientific KK, Tokyo, Japan). Fluorescent images were acquired using a wide-field fluorescence microscope (BZ-X810; Keyence). The cross-sectional areas (CSA) of individual muscle fibers were measured in three independent animals. For each muscle sample, the CSA values of individual fibers were averaged to obtain a single biological replicate.

### Myotube contraction assay

Fully differentiated C2C12 myotubes were stimulated to contract using electric pulse stimulation (BPSD-1001 CM; Uchida Denshi, Tokyo, Japan) under the following conditions: 1 Hz frequency, 30 ms pulse width (3% duty cycle), 100 mA current, and 50 V voltage. The relative contractility of C2C12 myotubes was quantified using a modified optical flow analysis implemented with the FlowJ plugin in ImageJ software (version 1.53q; National Institutes of Health, Bethesda, MD, USA). FlowJ, originally developed for object motion detection in video analysis, generates an optical flow field represented as a vector map indicating the displacement of individual pixels, which was used to assess myotube movement during contraction.

### TMT-based quantitative proteomics

Gastrocnemius muscle tissues were homogenized in radioimmunoprecipitation assay buffer supplemented with protease inhibitors and sonicated. The homogenates were centrifuged at 21,500 × *g* for 40 min at 4°C, and the supernatants were collected. Protein concentrations were determined using the bicinchoninic acid assay. Extracted proteins were reduced, alkylated, and digested with trypsin using the EasyPep Mini MS Sample Prep Kit (Thermo Fisher Scientific) according to the manufacturer’s instructions. The resulting peptides were labeled with tandem mass tags (TMT) using the TMT Mass Tagging Kit (Thermo Fisher Scientific) following the manufacturer’s protocol. Combined peptide samples were fractionated by high-pH reversed-phase chromatography (Thermo Fisher Scientific), dried, and reconstituted in 0.1% formic acid.

Liquid chromatography–tandem mass spectrometry (LC–MS/MS) was performed using an Orbitrap Explorer 480 and Orbitrap Fusion mass spectrometers (Thermo Fisher Scientific). Peptides were separated on a C18 nanoLC column (75 µm × 25 cm, 2 µm particle size) at a flow rate of 300 nL/min using a linear gradient of 5% to 35% solvent B (0.1% formic acid in acetonitrile) over 90 min, with solvent A being 0.1% formic acid in water. Data were acquired in data-dependent acquisition mode. Raw data were processed using Proteome Discoverer (version 3.1; Thermo Fisher Scientific). Intensity values were log-transformed and quantile normalized to reduce technical variance and ensure comparability across samples.

Principal component analysis (PCA) was performed using the prcomp function in R (version 4.5.1) on normalized data. Gene Set Enrichment Analysis (GSEA) was conducted in R using the ClusterProfiler package (version 4.16.0). Proteins lacking Entrez IDs were excluded prior to analysis. GSEA was performed using the gseKEGG function with the Kyoto Encyclopedia of Genes and Genomes database. The Benjamini–Hochberg method was applied to control the false discovery rate (FDR) for multiple hypothesis testing. Enriched pathways with a normalized enrichment score and an adjusted p-value (FDR) below 0.1 were considered statistically significant.

### Western blotting

The extracted protein samples were diluted in Laemmli sample buffer and boiled at 98°C for 10 min. Proteins were separated by 10% sodium dodecyl-sulfate polyacrylamide gel electrophoresis and transferred onto polyvinylidene fluoride membranes (Millipore, Bedford, MA, USA). Membranes were blocked with 5% skim milk in Tris-buffered saline containing Tween 20 (TBST; 20 mM Tris–HCl [pH 7.6], 137 mM NaCl, 0.1% Tween-20) for 1 h at 22°C–25°C, followed by overnight incubation at 4°C with primary antibodies. After washing with TBST, membranes were incubated for 1 h at 23°C with horseradish peroxidase (HRP)-conjugated secondary antibodies (anti-rabbit or anti-mouse IgG; 1:1000). All primary and secondary antibodies used for western blotting were purchased from Cell Signaling Technology (Danvers, MA, USA). Protein bands were visualized using an enhanced chemiluminescence substrate (Luminata Forte Western HRP Substrate; Merck Millipore) and imaged with the ImageQuant LAS 500 system (GE Healthcare, Amersham, UK).

### Lipidomic analyses

Total lipids were extracted from the gastrocnemius muscle, plasma, liver, and protein powder samples using chloroform/methanol (2:1, v/v), and lipid fractions were prepared following the Bligh and Dyer method. The extracted lipids were reconstituted in methanol and analyzed using thin-layer chromatography (TLC) and LC–MS on an Orbitrap 120 mass spectrometer (Thermo Scientific) equipped with an electrospray ionization source operated in both positive and negative ion modes. Chromatographic separation was performed on an Accucore C30 column (Thermo Scientific) maintained at 40°C with a flow rate of 0.25 mL/min. Solvent A consisted of 10 mM ammonium formate and 0.1% formic acid in acetonitrile/water (60:40, v/v), whereas solvent B comprised 10 mM ammonium formate and 0.1% formic acid in 2-propanol/water/acetonitrile (85:10:5, v/v/v). The gradient program was as follows: 30% B for 5 min, increased to 70% B for 5–14 min, ramped to 99% B for 14–21 min, and held for 3 min. The system was returned to the initial conditions within 0.1 min and equilibrated for 4 min. The injection volume was 2 µL. Lipid identification and quantification were performed using LipidSearch 5.0 (Mitsui Knowledge Industry, Tokyo, Japan), by matching MS/MS spectra to a curated lipid database and aligning features across samples for relative quantification.

### MS imaging

Frozen gastrocnemius muscle sections were mounted on indium tin oxide-coated glass slides and dried in a desiccator for 30 min. Tissue sections were uniformly coated with a matrix solution of 2,5-dihydroxybenzoic acid (50 mg/mL in methanol/water, 8:2, v/v) using a TM Sprayer 3.0 (HTX Technologies, Chapel Hill, NC, USA). Matrix-assisted laser desorption/ionization MS imaging was performed using a timsTOF fleX mass spectrometer (Bruker Daltonics, Billerica, MA, USA) equipped with a smartbeam 3D laser operating at 10 kHz. Analyses were conducted in positive ion mode over an m/z range of 400–1,200. The spatial resolution was set to 70 µm. Ion images were reconstructed using SCiLS Lab software (Bruker Daltonics) with root mean square normalization.

### Fatty acid composition analyses by gas chromatography–MS

The fatty acid composition of casein and APP powders was analyzed using gas chromatography–MS (GC–MS; Agilent 7890B GC coupled to a 5977B MSD; Agilent Technologies, Santa Clara, CA, USA). Total lipids were extracted from 0.1 g of powder using chloroform/methanol (2:1, v/v). The extracted lipids were subsequently methylated using a fatty acid methylation kit (Nacalai Tesque) according to the manufacturer’s instructions. The resulting fatty acid methyl esters (FAMEs) were reconstituted in 1 mL hexane, and 1 μL was injected in split mode (split ratio 10:1) onto a DB-Fast-FAME capillary column (30 m × 0.25 mm i.d., 0.25 μm film thickness; Agilent Technologies, Santa Clara, CA, USA). The oven temperature program was as follows: initial temperature of 50°C for 0.5 min, ramped to 175°C at 40°C/min, to 215°C at 15°C/min, and to 240°C at 5°C/min, where it was held for 5 min. The MS was operated in the electron ionization mode at 70 eV. The ion source and quadrupole temperature were set at 230°C and 150°C, respectively. Quantification was performed using calibration curves generated with an external standard (Supelco 37 Component FAME Mix; Merck, Darmstadt, Germany). Data acquisition and processing were performed using MassHunter software (Agilent Technologies).

### GLP-1 detection and DPP-4 activity assay

GLP-1 concentrations were measured using GLP-1 ELISA kit Wako, high sensitive (Fujifilm, 299−75501) according to the protocol. Plasma with DPP-4 inhibitor were used for the study. The measurement of DPP-4 activity was performed according to previously described methods [12,13] using serum collected without DPP-4 inhibitor. Briefly, the assay employed p-nitroaniline (4-nitroaniline, N2128, Sigma; MW 138.13) as the standard, Gly-Pro p-nitroaniline (G2901, Sigma; MW 464.49) as the substrate, 0.25 M Tris (2-amino-2-hydroxymethyl-1,3-propanediol; 207−06275, Wako) as the assay buffer, and 1 M acetic acid solution as the stop solution. The reaction was initiated by adding the p-nitroaniline standards to the standard wells or 80 µL of substrate solution to the sample and blank wells, after which the plate was incubated at 37°C for 60 min. The reaction was terminated by adding 40 µL of stop solution to all wells, followed by the addition of 5 µL of plasma to the blank wells. DPP-4 activity was calculated based on the amount of p-nitroaniline released from the substrate Gly-Pro p-nitroaniline. One unit of enzyme activity was defined as the amount of enzyme that releases 1 µmol of p-nitroaniline per min.

### Statistical analysis

Data are presented as the mean ± standard error of the mean. Statistical analyses were performed using GraphPad Prism 10 (GraphPad Software, La Jolla, CA, USA). Data normality was assessed using the Shapiro–Wilk test. For normally distributed data, Student’s *t*-test was applied to compare the casein- and APP-fed groups. For datasets that did not meet the assumption of normality, the Mann–Whitney U test was employed. The effects of protein source (casein vs APP) and time after ingestion were analyzed using two-way analysis of variance (ANOVA). When a significant interaction was detected, post hoc multiple comparisons were performed using Šidák’s correction to compare protein sources at each time point. Statistical significance was set at p < 0.05.

## Results

### APP feeding induced significant hypertrophy of fast-twitch muscle fibers

**Fig 1:**
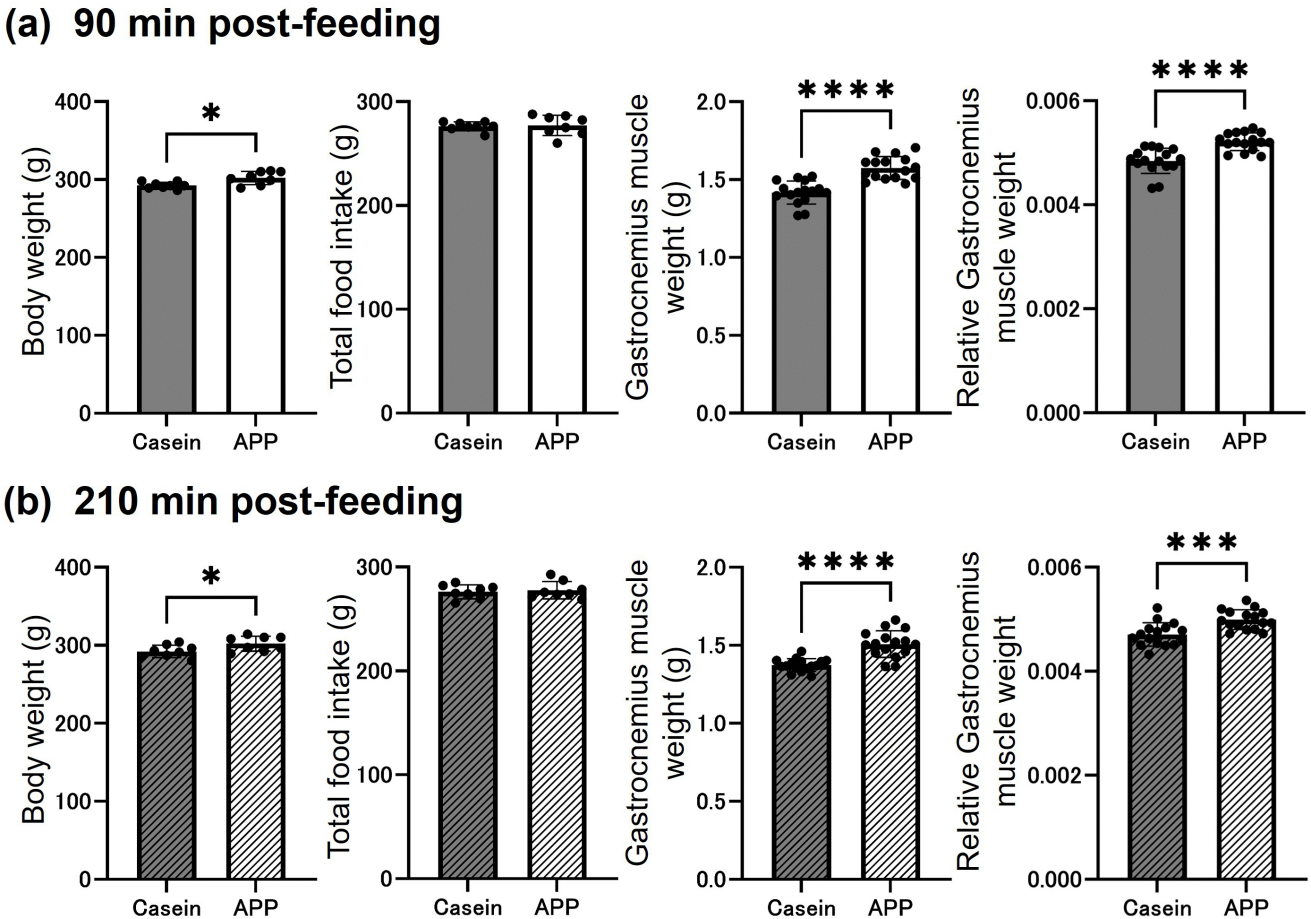
Comparison of body weight and gastrocnemius muscle mass between casein- and APP-fed rats. (a) Results at 90 and (b) 210 min post-feeding. From left to right, panels show final body weight, total food intake, gastrocnemius muscle weight, and body weight–normalized gastrocnemius muscle weight. Data are presented as mean ± SEM. Statistical comparisons were performed using Student’s *t*-test (N = 8). *; p < 0.05, ****; p < 0.0001.

**Fig 2:**
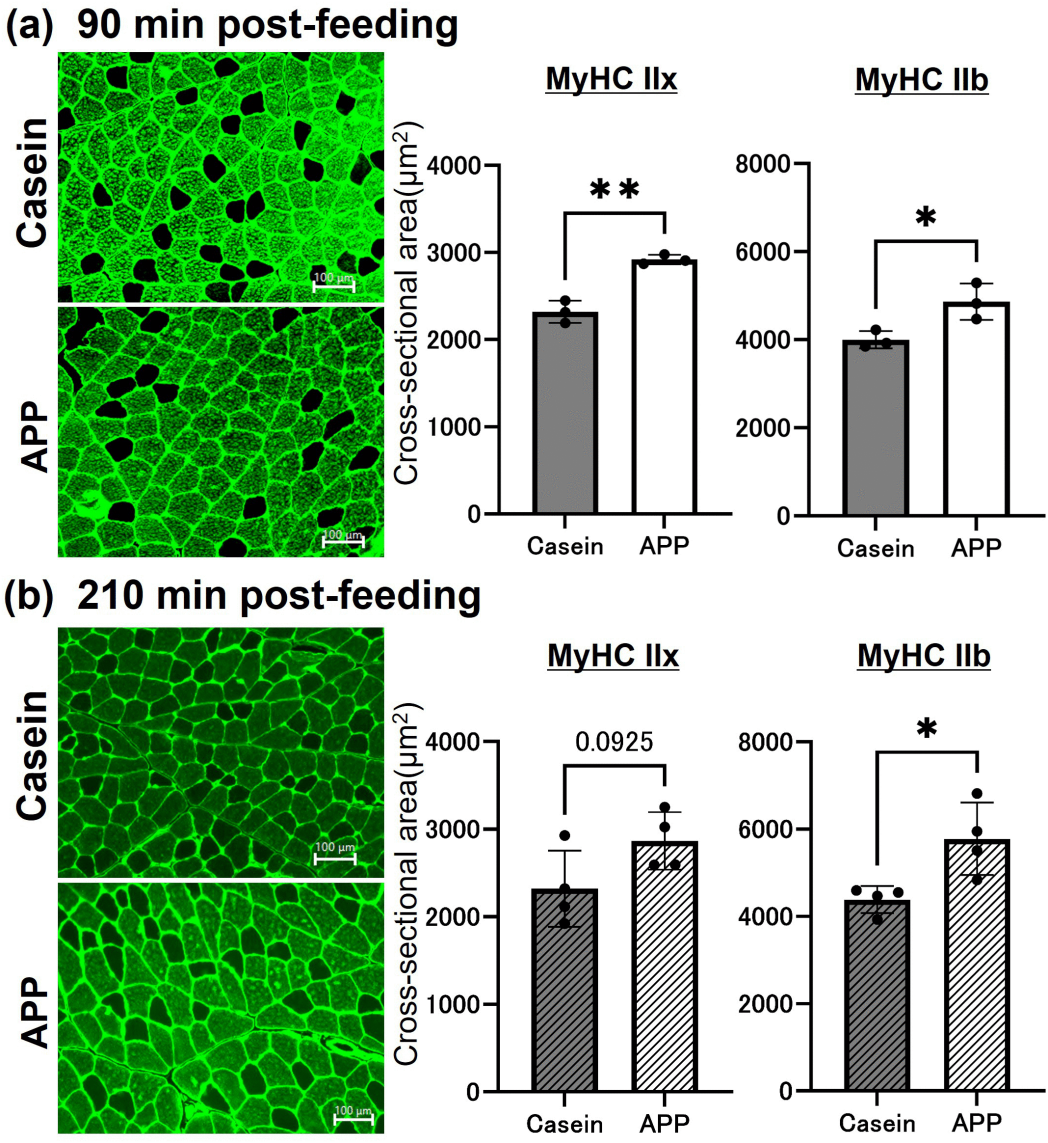
Comparison of gastrocnemius muscle cross-sectional area at different time points post-feeding. Immunofluorescence staining of the superficial region of the gastrocnemius muscle at (a) 90 and (b) 210 min post-feeding. Black indicates MyHC IIx–positive fibers, and green indicates MyHC IIb–positive fibers. The cross-sectional area of each fiber was calculated, and data are presented as the mean ± SEM. Statistical comparisons were performed using Student’s *t*-test (90 min: N = 3; 210 min: N = 4). *; p < 0.05, **; p < 0.01. Scale bar = 100 μm.

After the 2-week feeding period, final body weight, total food intake, and gastrocnemius muscle weight were evaluated. Both final body weight and gastrocnemius muscle weight were significantly higher in the APP group at 90 and 210 min postprandially. In addition, body weight–normalized gastrocnemius muscle weight was also significantly higher in the APP group. In contrast, total food intake did not differ between the groups (Fig 1). Fluorescence immunostaining was used to identify myofiber types (MyHC IIb, IIa, and IIx) and quantify the CSA of individual fibers in the superficial gastrocnemius region (Fig 2). The CSA of MyHC IIb fibers was significantly greater in the APP group than in the controls, and MyHC IIx fibers showed a significant increase at 90 min postprandially. We also stained and quantified the CSA of MyHC IIa fibers in the deep region; however, no changes were observed (Supplementary Figure 1). These results indicate that APP feeding promotes hypertrophy of fast-twitch fibers in the gastrocnemius muscle.

### Plasma treatment promoted myotube proliferation and contractile activity

**Fig 3:**
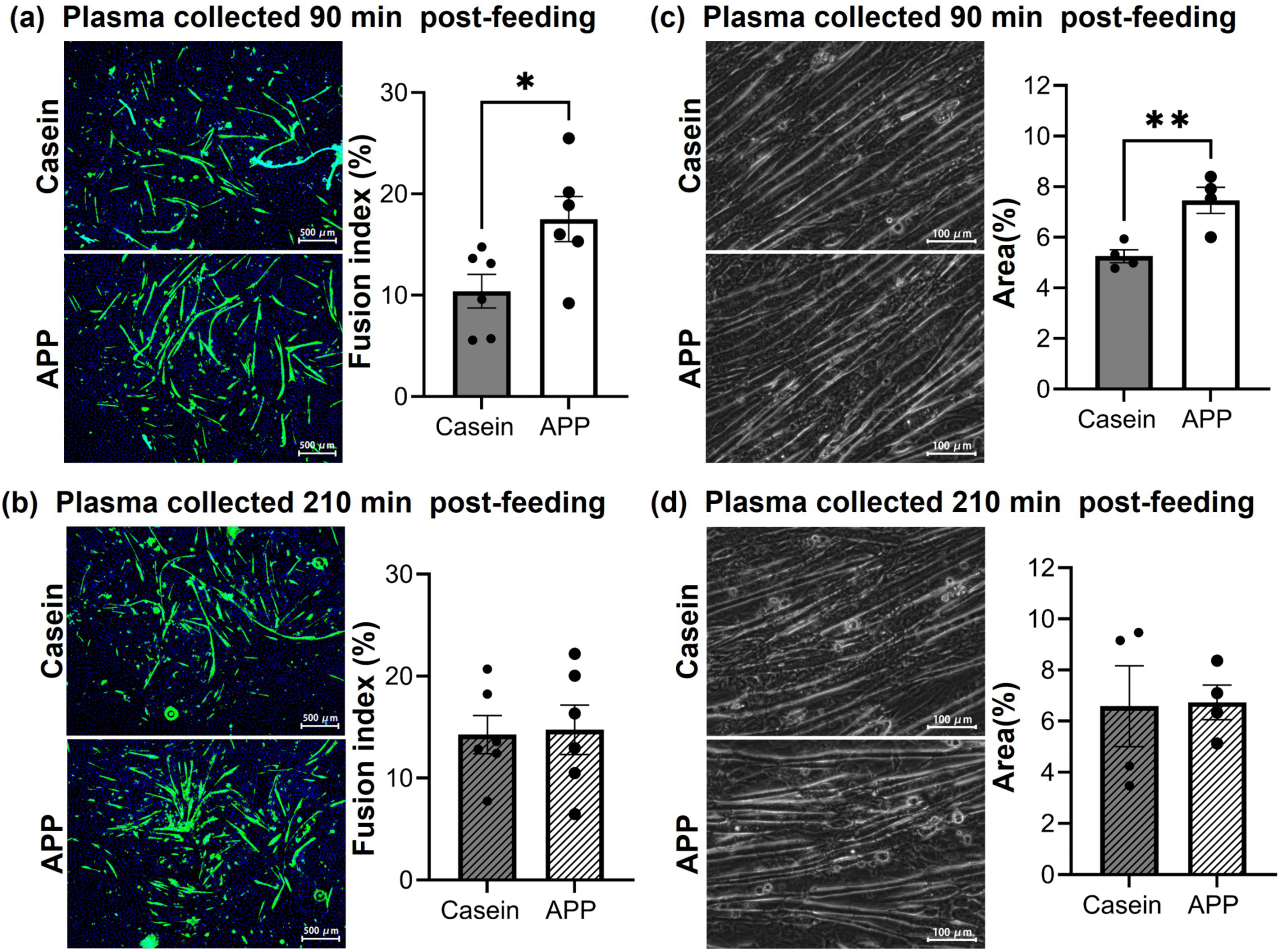
Fusion index and muscle contractile activity following treatment with rat plasma collected at different time points post-feeding. Cells were differentiated, then treated for 48-72 h with 0.5% plasma collected at 90 or 210 min after feeding, supplemented into the differentiation medium. (a, b) Fusion index values were quantified from immunofluorescence images (N = 6). (c, d) Contractile areas were quantified using FlowJ (N = 4). Data are presented as mean ± SEM. Statistical significance was determined using Student’s *t*-test. *; p < 0.05, **; p < 0.01.

The effects of plasma derived from rats in the synchronized feeding model on C2C12 myotubes were evaluated (Fig 3). Myotubes were incubated with 0.5% rat plasma in differentiation medium for 72 h, followed by immunofluorescence staining for sarcomeric MyHC to calculate the fusion index and assess differentiation (Fig 3a, 3b). The fusion index was significantly higher in the APP group when the plasma was collected 90 min after feeding, whereas no change was observed with plasma collected at 210 min. To assess contractility, C2C12 myotubes were treated with 0.5% plasma for 48 h, then subjected to electrical stimulation (Fig 3c, 3d). Plasma collected 90 min after APP feeding significantly increased the contractile area compared with that of casein feeding, whereas no difference was observed between groups at 210 min. These findings suggest that plasma collected 90 min after APP feeding contains factor(s) that promote myotube hypertrophy and enhance contractile function.

### Measurements of GLP-1 concentration and DPP-4 activity

**Fig 4:**
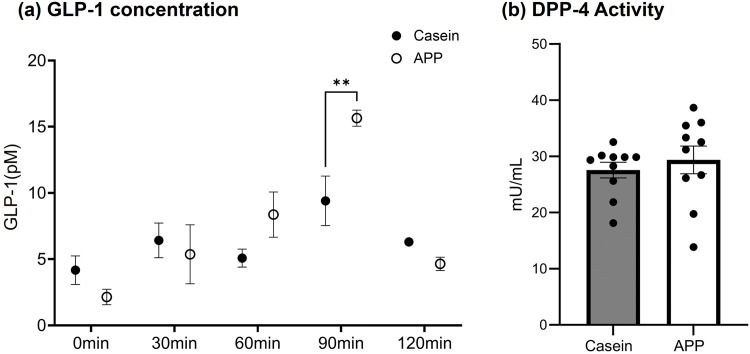
Measurements of GLP-1 levels and DPP-4 activity. (a) Results of GLP-1 concentration measurements in plasma. Data are shown as individual values with mean ± SEM. Statistical analysis was performed using two-way ANOVA (protein source × time), followed by Šidák’s multiple-comparisons test to compare protein sources at each time point (N = 4). **p < 0.01 (Casein vs APP at 90 min). (b) Results of the serum DPP-4 activity assay. Statistical significance was evaluated using the Mann–Whitney U test (N = 10).

Based on the *in vitro* findings shown in Fig 3, we hypothesized that plasma contained physiological factors that contributed to skeletal muscle hypertrophy. On the basis of previous studies demonstrating reduced blood glucose levels after APP feeding [14,15], we measured GLP-1 concentrations in plasma (Fig 4a). Plasma samples were collected at multiple time points after feeding to evaluate time-dependent changes in circulating GLP-1 levels. Two-way ANOVA revealed a significant main effect of time and a significant interaction between protein source and time, whereas the main effect of protein source was not significant. Given the significant interaction, post hoc multiple comparisons were performed using Šidák’s correction to compare protein sources at each time point. Plasma GLP-1 concentrations were significantly higher in the APP-fed group than in the casein-fed group at 90 min after feeding, whereas no significant differences between groups were observed at the other time points (Fig 4a). Because circulating GLP-1 levels can be influenced by enzymatic degradation mediated by DPP-4, we next measured serum DPP-4 activity. No significant difference in DPP-4 activity was detected between the casein- and APP-fed groups (Fig 4b).

### Proteomic analysis of the gastrocnemius muscle revealed alterations in metabolic status

**Fig 5:**
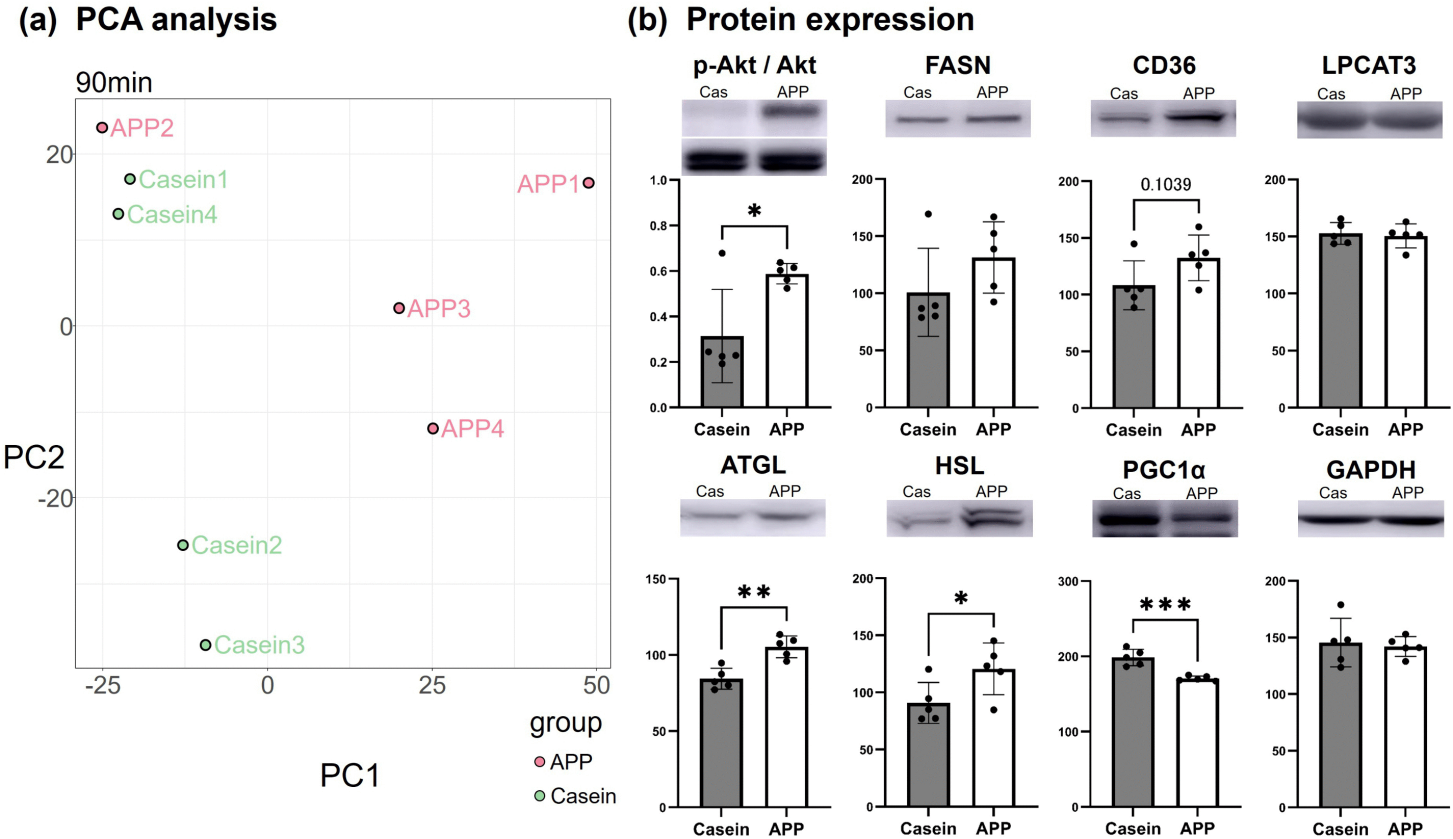
TMT-based proteomic analysis of rat gastrocnemius muscle 90 min post-feeding. (a) Results of PCA from the TMT-based proteomic analysis. (b) Western blot and quantification of lipid metabolism-related proteins. Akt: protein kinase B, p-Akt: phosphorylated Akt, FASN: fatty acid synthase, CD36: platelet glycoprotein 4, LPCAT3: lysophosphatidylcholine acyltransferase 3, ATGL: adipose triglyceride lipase, HSL: hormone-sensitive lipase, PGC1α: peroxisome proliferator-activated receptor gamma coactivator 1-alpha, GAPDH: glyceraldehyde-3-phosphate dehydrogenase. Data are presented as mean ± SEM. Statistical significance was determined using Student’s *t*-test. (N = 5). *; p < 0.05, **; p < 0.01, ***; p < 0.001.

Considering the hypertrophic effects of plasma collected 90 min after feeding, we performed a TMT-based proteomic analysis of gastrocnemius muscle harvested at the same time point. PCA revealed a clear separation of protein expression profiles between the casein and APP groups (Fig 5a). In total, 2,302 proteins were identified and quantified [16]. Pathway enrichment analysis showed significant upregulation of the PPAR signaling pathway, fatty acid metabolism and degradation pathways, peroxisomal pathway, oxidative phosphorylation, motor proteins, and arginine biosynthesis, suggesting enhanced lipid utilization, mitochondrial activity, and energy supply for contractile functions. In contrast, proteasomes, insulin signaling, ribosomes, and disease-related modules such as spinocerebellar ataxia were significantly downregulated. These findings indicate a transient suppression of protein translation, protein degradation, and insulin-dependent anabolic signaling, accompanied by a shift toward fatty acid–driven energy metabolism (Table 1). To further assess lipid metabolism-related proteins, we performed western blotting. Phosphorylated Akt (AKT serine/threonine kinase) was markedly increased, indicating activation of Akt–mTOR signaling following APP supplementation. The expression levels of fatty acid synthase (FASN), CD36, and lysophosphatidylcholine acyltransferase 3 (LPCAT3) remained unchanged, whereas adipose triglyceride lipase (ATGL) and hormone-sensitive lipase (HSL) were significantly upregulated in the APP group. In contrast, peroxisome proliferator-activated receptor gamma coactivator 1-alpha (PGC1α) expression was significantly reduced (Fig 5b).

**Table 1:** Significantly enriched pathways identified by GSEA of proteomic data following APP feeding.

ID	Description	setSize	NES	Changes	p.adjust	qvalue
rno03320	PPAR signaling pathway	15	2.139	Up	0.0042	0.0040
rno00071	Fatty acid degradation	16	2.123	Up	0.0061	0.0057
rno01212	Fatty acid metabolism	15	2.073	Up	0.0066	0.0062
rno04146	Peroxisome	15	2.027	Up	0.0134	0.0125
rno00190	Oxidative phosphorylation	46	1.842	Up	0.0438	0.0409
rno04814	Motor proteins	23	1.825	Up	0.0922	0.0861
rno00220	Arginine biosynthesis	6	1.778	Up	0.0702	0.0656
rno03050	Proteasome	17	−2.119	Down	0.0066	0.0062
rno04910	Insulin signaling pathway	21	−1.969	Down	0.0438	0.0409
rno03010	Ribosome	25	−1.926	Down	0.0438	0.0409
rno05017	Spinocerebellar ataxia	23	−1.794	Down	0.0985	0.0920

Proteomic data were analyzed by GSEA using the KEGG database. Significantly enriched pathways are presented with their normalized enrichment scores and adjusted p-values (FDR < 0.1).

### Lipidomic analysis revealed selective upregulation of docosahexaenoic acid-containing phosphatidylcholines in hypertrophied muscle

**Fig 6:**
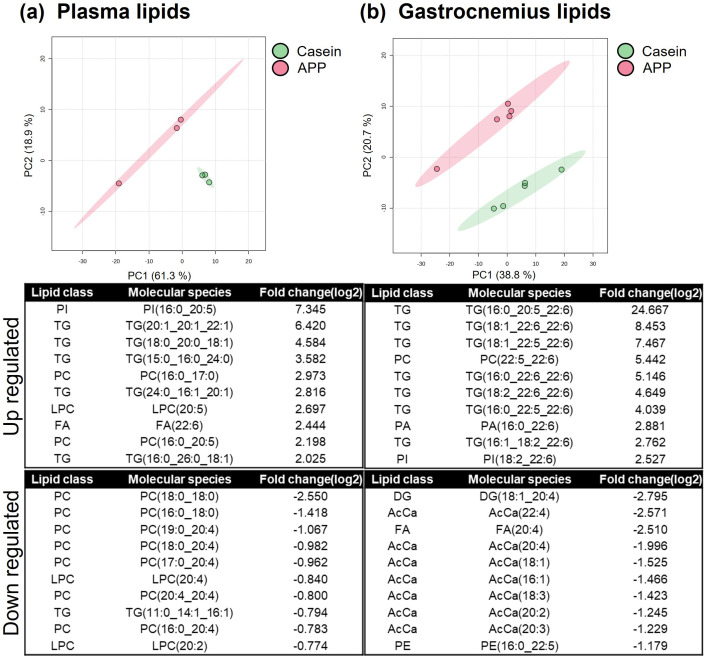
Lipidomic profiling of rat plasma and gastrocnemius muscle at 90 min post-feeding. LC–MS data were analyzed using LipidSearch 5.0. Comparisons between the casein- and APP-fed groups are shown for (a) plasma and (b) gastrocnemius muscles. The upper panels show the PCA results, while the lower panels present the top 10 upregulated and downregulated molecular species. All molecules shown exhibited significant differences. Fold change and p-value thresholds were set at 1.5 and 0.05, respectively. AcCa: Acylcarnitine, FA: Fatty Acid, PA: Phosphatidic Acid, PC: Phosphatidylcholine, LPC: Lysophosphatidylcholine, PE: Phosphatidylethanolamine, PI: Phosphatidylinositol, TG: Triacylglycerol, DG: Diacylglycerol.

**Fig 7:**
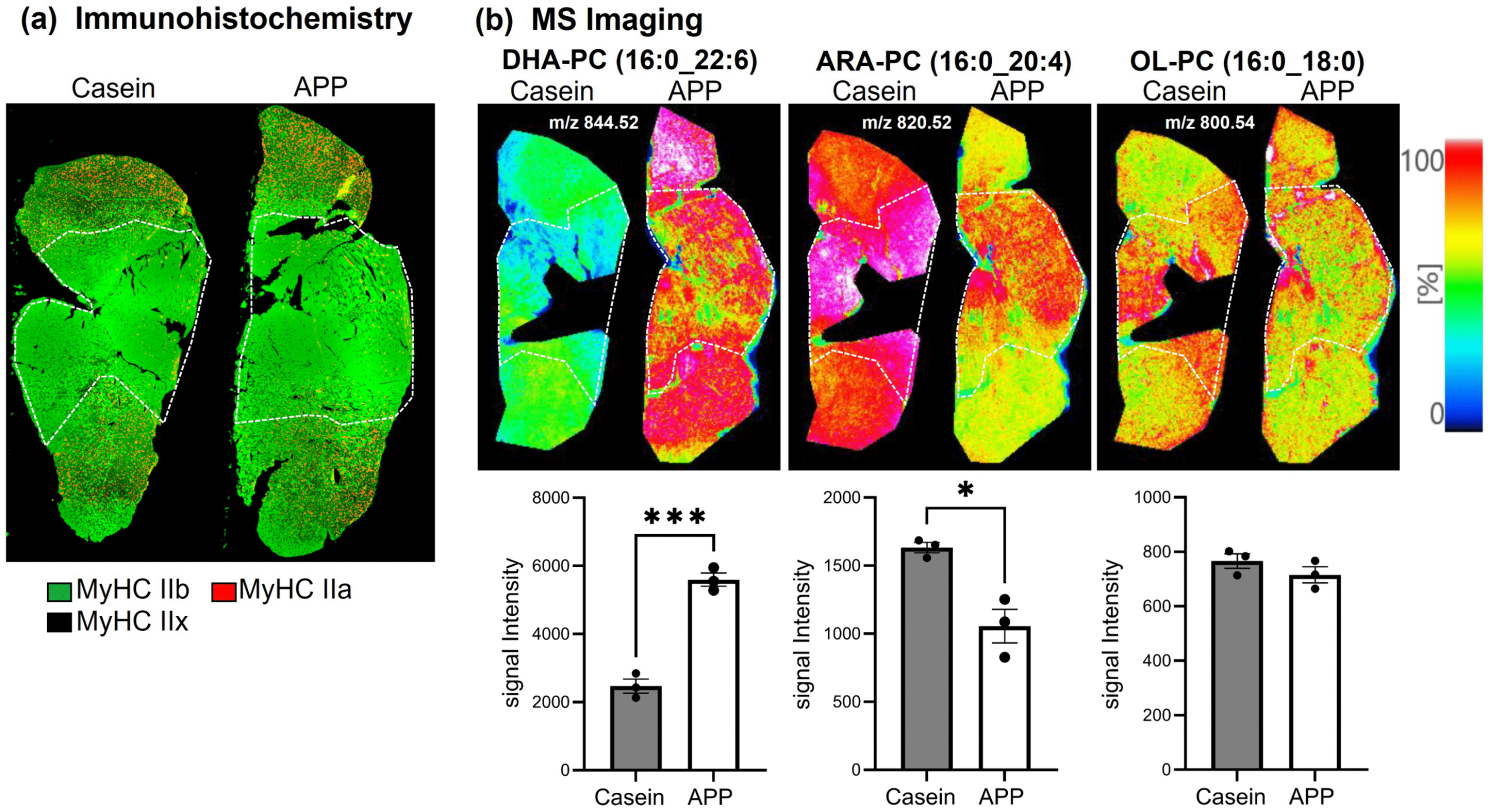
Mass spectrometry imaging of phosphatidylcholine in gastrocnemius muscle tissue. (a) Immunofluorescence images using MHC Type IIa and IIb-specific antibodies delineate fast-twitch fiber-dominated regions, indicated by white dotted lines. (b) Ion images of DHA-PC (m/z 844.52), ARA-PC (m/z 820.52), and OL-PC (m/z 800.54) are shown, and their signal intensities (white dotted area) were quantified. *; p < 0.05, ***; p < 0.001.

**Fig 8:**
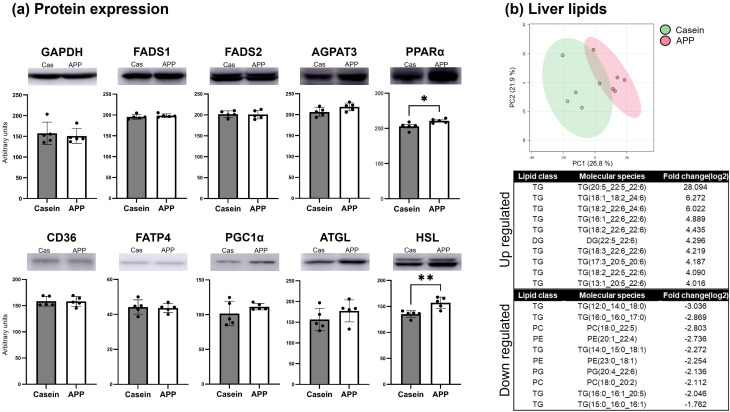
Protein expression and lipid alterations in liver tissue. (a) Western blot and quantification of lipid metabolism–related proteins in liver tissue. FADS1: fatty acid desaturase 1, FADS2: fatty acid desaturase 2, AGPAT3: 1-acylglycerol-3-phosphate O-acyltransferase 3, PPARα: Peroxisome proliferator-activated receptor alpha, FATP4: fatty acid transport protein 4. Data are presented as mean ± SEM (N = 5). Statistical significance was determined using unpaired Student’s *t*-test. **; p < 0.01. (b) Lipidomic analysis of liver tissue performed with LipidSearch 5.0. The upper panels show the PCA, and the lower panels display the top 10 significantly upregulated and downregulated molecular species. Thresholds for fold change and p-value were set at 1.5 and 0.05, respectively. PC: Phosphatidylcholine, PE: Phosphatidylethanolamine, PG: Phosphatidylglycerol, TG: Triacylglycerol, DG: Diacylglycerol.

To further investigate lipid metabolism, lipidomic analysis of plasma and skeletal muscle was performed using LC–MS, identifying 284 lipid species in plasma and 538 in skeletal muscle (Fig 6). PCA showed a clear separation between the casein and APP groups. In plasma, statistical analyses demonstrated a significant increase of FA 22:6 (docosahexaenoic acid; DHA) in the APP-fed group (Fig 6a). Similarly, DHA-containing phospholipids and neutral lipids were significantly enriched in skeletal muscle (Fig 6b). In contrast, lysophosphatidylcholines (LPC) containing arachidonic acid (ARA; 20:4) and ARA-containing phosphatidylcholines (PCs), which serve as precursors for bioactive lipid mediators involved in the regulation of inflammatory and regenerative processes, were decreased in both plasma and muscle. Acylcarnitines were also decreased in muscle by APP. To localize these lipids within skeletal muscle, MS imaging was performed on gastrocnemius cryosections combined with immunofluorescence to identify the superficial region enriched in fast-twitch fibers (MyHC IIx and IIb; Fig 7a). Ions corresponding to the potassium-adduct DHA-containing PC (DHA-PC; *m/z* 844.52), ARA-PC (*m/z* 820.52), and oleic acid (OL)-PC (*m/z* 800.54) were analyzed (Fig 7b). OL-PC showed no difference between groups, whereas DHA-PC accumulated markedly in hypertrophied fast-twitch fibers, accompanied by a reduction in ARA-PC. Notably, similar trends in DHA-PC accumulation and ARA-PC reduction were also observed in slow-twitch regions; however, no hypertrophic changes were observed in slow-twitch fibers. As circulating lipid composition is influenced by hepatic metabolism, hepatic enzymes were examined by western blotting (Fig. 8a). The expression levels of fatty acid desaturase 1 (FADS1), fatty acid desaturase 2 (FADS2), and 1-acylglycerol-3-phosphate O-acyltransferase 3 (AGPAT3; enzymes involved in DHA-PC synthesis), as well as CD36 and long-chain fatty acid transport protein 4 (FATP4; DHA transporters), showed no significant change. Similarly, PGC1α expression was unaltered. In contrast, the expression level of peroxisome proliferator-activated receptor alpha (PPARα) showed a significant increase in the APP-fed group. Consistent with plasma and muscle findings, lipidomic analysis of liver tissue also revealed a significant increase in DHA-containing lipids (Fig 8b). Although the expression of DHA-synthesizing enzymes remained unchanged, HSL was significantly upregulated, contributing to neutral lipid breakdown. These findings suggest that APP supplementation enhances neutral lipid degradation.

**Fig 9:**
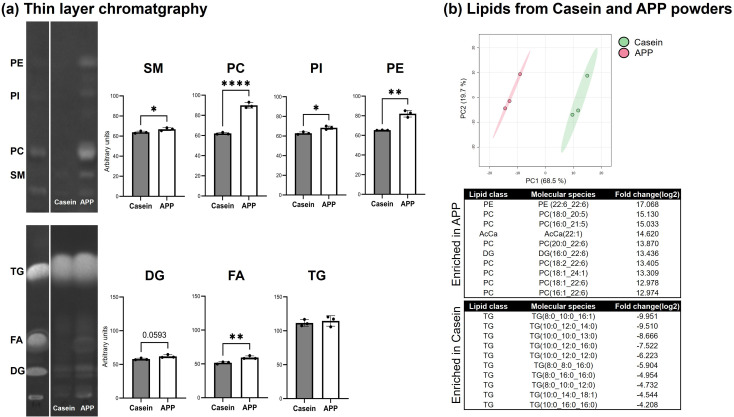
Lipidomic analyses of protein powders. (a) Thin-layer chromatography of extracted lipids from casein and APP powders. Statistical significance was determined using Student’s *t*-test. (N = 3). *; p < 0.05, **; p < 0.01, ***; p < 0.001. (b) Lipidomic analysis of protein powders performed with LipidSearch 5.0. The upper panels show the PCA results. The table summarizes the top 10 molecular species that were significantly enriched in APP powder (upper) and casein powder (lower). Thresholds for fold change and p-value were set at 1.5 and 0.05, respectively. AcCa: Acylcarnitine, PC: Phosphatidylcholine, PE: Phosphatidylethanolamine, TG: Triacylglycerol, DG: Diacylglycerol.

To assess the dietary contribution of DHA, the intrinsic lipid content of the protein powders was analyzed by GC–MS, TLC, and LC–MS. Both diets contained 7% soybean oil as the primary lipid source, and the total lipid content of the protein powders (134.1–223.6 mg lipids per 100 g of feed) did not differ significantly (Table 2). APP powder contained higher amounts of monounsaturated fatty acids (C16:1, C20:1, C22:1, and C24:1) and polyunsaturated fatty acids, including DHA, eicosapentaenoic acid (EPA), and ARA. Notably, the DHA content was only 0.008% of feed (8.47 mg/100 g feed), which is a negligible amount (Table 2). Although the amounts of neutral lipids did not differ between the two powders, phospholipid levels were higher in APP than in casein (Fig 9a). LC–MS analysis clearly demonstrated that APP powder contained significantly higher levels of DHA-containing phospholipids than did casein powder, while neither powder contained DHA within the triglyceride fraction. In contrast, casein powder contained significantly higher levels of triglycerides with mid-chain fatty acids (Fig 9b).

**Table 2:** Contents of individual fatty acids (µg) in the protein portion per 10 g of feed.

Fatty acids	Sample	Mean±SEM(μg)	Fatty acids	Sample	Mean±SEM(μg)	Fatty acids	Sample	Mean±SEM(μg)
**C8**	Casein	0.10 ± 0.01	**C18:1t**	Casein	21.06 ± 21.06	**C20:3n6**	Casein	0.31 ± 0.05
	APP	0.12 ± 0.02		APP	3.41 ± 1.25		APP	3.12 ± 2.60
**C10**	Casein	0.22± 0.05†	**C18:1c**	Casein	2303.03 ± 723.63	**C20:4**	Casein	0.35 ± 0.12
	APP	0.07 ± 0.02		APP	3970.35 ± 419.12		APP	25.98 ± 4.12**
**C11**	Casein	0.37 ± 0.22	**C18:2t**	Casein	1.45 ± 0.89	**C22**	Casein	71.63 ± 27.78
	APP	0.08 ± 0.02		APP	4.44 ± 1.00†		APP	135.53 ± 13.31
**C12**	Casein	9.30 ± 3.40†	**C18:2c**	Casein	6003.01 ± 1010.89	**C20:5**	Casein	1.70 ± 0.63
	APP	0.98 ± 0.10		APP	7884.07 ± 344.14		APP	534.75 ± 55.92***
**C14**	Casein	55.09 ± 19.79	**C18:3n3**	Casein	120.74 ± 38.68	**C22:1**	Casein	1.06 ± 0.32
	APP	93.93 ± 9.04		APP	247.27 ± 28.00†		APP	3.09 ± 0.616*
**C14:1**	Casein	3.37 ± 1.28	**C18:3n6**	Casein	1459.56 ± 347.17	**C22:2**	Casein	0.70 ± 0.258
	APP	1.35 ± 0.32		APP	2318.85 ± 16.78†		APP	0.23 ± 0.08
**C16:0**	Casein	2264.61 ± 764.91	**C20**	Casein	68.11 ± 25.76	**C23**	Casein	6.33 ± 2.43
	APP	4148.91 ± 286.49†		APP	130.3 ± 11.76†		APP	11.93 ± 1.19
**C16:1**	Casein	18.96 ± 5.62	**C20:1**	Casein	41.98 ± 17.09	**C24**	Casein	21.12 ± 8.618
	APP	64.82 ± 7.67**		APP	112.82 ± 7.46*		APP	39.9 ± 3.79
**C17**	Casein	20.72 ± 7.80	**C20:2**	Casein	4.6 ± 1.69	**C24:1**	Casein	0.19 ± 0.043
	APP	42.39 ± 3.94†		APP	8.04 ± 0.81		APP	6.56 ± 0.775**
**C17:1**	Casein	15.83 ± 7.88	**C21**	Casein	4.56 ± 1.68	**C22:6**	Casein	0.42 ± 0.04
	APP	36.89 ± 11.73		APP	8.05 ± 0.825		APP	847.51 ± 82.389***
**C18**	Casein	887.64 ± 328.97	**C20:3n3**	Casein	2.42 ± 0.72	**Total fatty acids**	Casein	13410.51 ± 3337.26
	APP	1671.24 ± 138.99†		APP	3.32 ± 1.18		APP	22360.29 ± 1594.40†

The fatty acid content of each powder was quantified by GC–MS. Based on the amounts incorporated into the rat diet (20 g casein powder or 18.45 g APP powder), fatty acid levels per 10 g of diet were calculated. Data are presented as mean ± SEM. Comparisons between groups were performed using Student’s *t*-test (N = 3). †; p < 0.1, *; p < 0.05, **; p < 0.01, ***; p < 0.001.

## Discussion

This study demonstrates that APP feeding promotes pronounced hypertrophy of fast-twitch fibers in the gastrocnemius muscle, accompanied by both systemic and tissue-specific remodeling of lipid metabolism. These effects occurred without changes in food intake or caloric provision, indicating that the observed remodeling was driven by the intrinsic properties of APP rather than by energy intake. Within 90 min, APP induced rapid proteomic changes in skeletal muscle and generated plasma factors that stimulated myotube hypertrophy *in vitro*. Plasma collected at 90 min promoted C2C12 hypertrophy, whereas plasma collected at 210 min did not, suggesting a transient peak of hypertrophic factors in circulation at 90 min. This interpretation is consistent with the marked increase in Akt phosphorylation observed at the same time point (Supplementary Figure 2), implying that the *in vitro* hypertrophic response was associated with the specific plasma milieu present at 90 min. Among circulating factors altered by APP feeding, GLP-1 exhibited a distinct time-dependent response rather than a sustained elevation. Plasma GLP-1 levels in APP-fed rats showed a transient increase, peaking at 90 min after feeding. Importantly, serum DPP-4 activity remained unchanged, indicating that the observed differences in GLP-1 levels primarily reflect altered secretion dynamics from intestinal L cells rather than changes in enzymatic degradation. Given that GLP-1 enhances glucose clearance by stimulating insulin secretion, this transient postprandial elevation is consistent with previous observations that APP-fed models exhibit lower blood glucose levels [14,15]. Moreover, our *in vitro* experiments showed that GLP-1 treatment induced myotube hypertrophy [17], supporting a potential role for GLP-1 signaling in skeletal muscle anabolic responses. However, because direct interference with GLP-1 signaling was not performed in the present study, a causal role for GLP-1 cannot be definitively established. Overall, these findings suggest that GLP-1 is likely one of several plasma-derived factors contributing to APP-induced skeletal muscle hypertrophy. Whether GLP-1 directly mediates skeletal muscle responses or acts indirectly via systemic signaling remains to be determined. Considering that protein hydrolysates from other fish species have already been reported to stimulate GLP-1 secretion in STC-1 cells [18], this suggests that the promotion of GLP-1 release represents a key physiological action of fish-derived proteins.

Proteomic analyses of muscle revealed activation of lipid metabolic pathways—including PPAR signaling, fatty acid metabolism, and peroxisomal activity—along with enhanced oxidative phosphorylation and reduced insulin signaling. These coordinated changes in protein expression may suggest a shift toward increased fatty acid utilization; however, this interpretation is based on pathway enrichment analyses rather than direct metabolic measurements. The concurrent suppression of ribosomal and proteasomal pathways suggests a temporary redistribution of cellular resources. Although Akt phosphorylation was enhanced at 90 min, indicating activation of anabolic signaling pathways, this early signaling response does not necessarily imply immediate changes in global protein synthesis. Consistent with these observations, long-term APP feeding resulted in skeletal muscle hypertrophy, confirming the subsequent enhancement of protein synthesis.

In addition to its skeletal muscle hypertrophic effects, APP selectively altered lipid handling in both the liver and muscle (Fig 5, 8). In skeletal muscle, APP feeding significantly increased the expression of the lipolytic enzymes ATGL and HSL, suggesting enhanced triglyceride hydrolysis. In contrast, in the liver only HSL expression was increased, whereas ATGL remained unchanged, indicating a distinct pattern of lipid regulation compared with muscle. Hepatic PPARα expression was also elevated in APP-fed rats; this may contribute to altered hepatic lipid metabolism and facilitate neutral lipid turnover. Collectively, these tissue-specific changes may be associated with the previously reported reduction in circulating lipids [7,15,19]. This process may facilitate fatty acid mobilization for metabolic use and contribute to remodeling of the systemic lipid milieu. Notably, in skeletal muscle, these changes were accompanied by a decrease in PGC1α expression (Fig 5) and reduced acylcarnitine levels (Fig 6), suggesting altered mitochondrial fatty acid handling and potential redistribution of liberated fatty acids toward structural lipid incorporation. This interpretation is supported by lipidomic and MS imaging analyses, which revealed selective enrichment of DHA-PC in hypertrophied fast-twitch fibers. Incorporation of DHA into membrane phospholipids has been implicated in skeletal muscle remodeling and functional adaptation [20]. DHA-PC has been reported to influence membrane fluidity, mitochondrial function, and anabolic signaling pathways relevant to muscle plasticity [20,21]. In this context, the selective accumulation of DHA-PC observed in hypertrophied fast-twitch fibers is consistent with adaptive membrane remodeling rather than increased dietary DHA supply. Conversely, ARA-PC serves as a precursor for eicosanoid signaling molecules that regulate inflammatory and regenerative processes in skeletal muscle [22]. Therefore, the observed reduction in plasma ARA-PC may reflect altered lipid signaling dynamics during muscle adaptation. Taken together, these findings suggest that coordinated changes in DHA-PC and ARA-PC are consistent with lipid remodeling processes accompanying fast-twitch fiber hypertrophy, rather than representing direct evidence of increased DHA biosynthetic flux. These lipid remodeling changes may contribute to a metabolic environment supportive of muscle adaptation. Although canonical activation of hepatic PPARα signaling could not be fully confirmed, APP may influence hepatic fatty acid metabolism by altering substrate utilization or intracellular lipid partitioning. These changes are consistent with the observed shift in fatty acid composition toward a higher DHA/ARA ratio in both plasma and skeletal muscle.

Although these findings highlight the prominent role of APP in lipid remodeling, it is important to note that the APP powder contained only trace amounts of DHA (0.008% of the diet). Trace DHA is consistently detected in fish-derived protein fractions [14,23], but the level detected in the present study was substantially lower than previously reported values. Based on our direct measurements, this dietary DHA level corresponds to approximately 1.6–1.7 mg DHA per day (20 g feed). Importantly, the experimental diet also contained 7% soybean oil, which provides a substantial amount of α-linolenic acid (ALA), a metabolic precursor of DHA. Because ALA typically accounts for approximately 6–7% of total fatty acids in soybean oil, this corresponds to approximately 84–98 mg ALA per day in 20 g of feed (https://www.feedtables.com/content/soybean-oil). The metabolic conversion of ALA to DHA generally occurs at an efficiency of approximately 2–5% under physiological conditions [24,25]. Thus, DHA generated endogenously from ALA may be comparable to the trace amount supplied by APP. Consistent with this interpretation, previous supplementation studies have shown that long-term intake of low-dose DHA (0.1% of diet for 10 weeks) does not significantly increase DHA content in skeletal muscle [26]. These findings suggest the existence of a threshold level of dietary DHA required to alter tissue lipid composition, as most studies investigating physiological effects of DHA supplementation employ dietary DHA levels exceeding 0.1% of diets [27]. Collectively, these findings indicate that the trace DHA present in APP is unlikely to exert substantial direct physiological effects and may be overshadowed by endogenous DHA synthesis from ALA. These observations suggest that bioactive components other than DHA are likely responsible for the APP-induced skeletal muscle hypertrophy observed in this study. Supporting this concept, a previous study comparing fish protein and fish oil intake demonstrated that the fish protein group, which contained only 0.02% DHA, exhibited significantly elevated plasma creatinine levels—a surrogate marker of increased skeletal muscle mass—whereas the fish oil group, containing substantially higher DHA levels (0.585%), did not show this effect [28]. These findings strongly suggest that the hypertrophic effect is attributable to the protein component of fish protein preparations rather than their DHA content. APP intake was associated with upregulation of HSL, which may promote mobilization of neutral lipid pools. HSL preferentially hydrolyzes substrates enriched in ARA rather than DHA [29], and released ARA is metabolized through eicosanoid pathways [30], leading to its depletion in circulation. Furthermore, our previous study showed that endurance training increases DHA-PC specifically in fast-twitch fibers without any change in DHA availability [31], supporting the idea that increased DHA-PC in hypertrophied muscle reflects muscle remodeling rather than the difference in dietary DHA input. The increase in DHA-PC likely reflects endogenous remodeling of membrane lipid composition rather than direct incorporation of dietary DHA. Acyltransferase enzymes have been suggested to contribute to DHA-PC accumulation [32] and remodeling during muscle hypertrophy [20], and future studies are warranted to examine these enzymes in our model.

Overall, our findings suggest that APP-induced hypertrophy may involve coordinated regulation of anabolic signaling and metabolic remodeling, rather than a single dominant mechanism. APP may function as a high-quality protein source that stimulates muscle protein synthesis and modulates lipid metabolic pathways. By enhancing lipolysis in the liver and muscle, APP further promotes lipid catabolism, reduces circulating triglycerides [19], and integrates these lipidomic and proteomic alterations to create a favorable metabolic environment that supports fast-twitch fiber hypertrophy, which is critical for locomotor performance and metabolic health. This study has several limitations. The mechanisms whereby APP selectively enriches DHA-containing phospholipids while limiting ARA incorporation remain unclear. Moreover, the present findings are based on rodent models and *in vitro* assays, and, thus, their direct translatability to humans remains uncertain. Future studies should aim to identify additional plasma mediators underlying the postprandial effects of APP, including the use of plasma fractionation approaches such as heat inactivation or selective lipid depletion to distinguish proteinaceous, lipid-derived, and hormonal contributors. Further studies are also required to clarify the molecular basis of DHA retention and to conduct translational investigations in humans to evaluate whether APP can be utilized to promote muscle growth and lipid remodeling for the prevention of sarcopenia and lifestyle-related diseases.

## Supporting information

S1 Fig Immunofluorescence staining of the deep region of gastrocnemius muscle. Immunofluorescence staining of the deep region of the gastrocnemius muscle at (a) 90 and (b) 210 min post-feeding. Black indicates MyHC IIx–positive fibers, green indicates MyHC IIb–positive fibers, and red indicates MyHC IIa-positive fibers. The cross-sectional area of red fibers was calculated, and data are presented as the mean ± SEM. Statistical comparisons were performed using Student’s t-test (90 min: N = 3; 210 min: N = 4). *; p < 0.05, **; p < 0.01. Scale bar = 100 μm.
(JPG)

S2 Fig Ratio of phosphorylated Akt to total Akt (pAkt/Akt) in skeletal muscle. The ratio of phosphorylated Akt to total Akt (pAkt/Akt) in skeletal muscle was measured at 0, 30, 60, 90, 120, and 210 min after feeding. Data are presented as the mean ± SEM. Two-way ANOVA revealed significant effects of time and diet, as well as a significant interaction between time and diet. Post hoc multiple comparisons using Sidak’s test indicated a significant difference between dietary groups at 90 min only. * * P < 0.01.
(JPG)
