# Supplementary figures and images for: Alaska pollock protein as a functional dietary source for promoting skeletal muscle hypertrophy and lipid metabolic remodeling
Source: PLoS One. 2026 May 13;21(5):e0348366. doi: 10.1371/journal.pone.0348366 (PMC13170884; doi:10.1371/journal.pone.0348366)

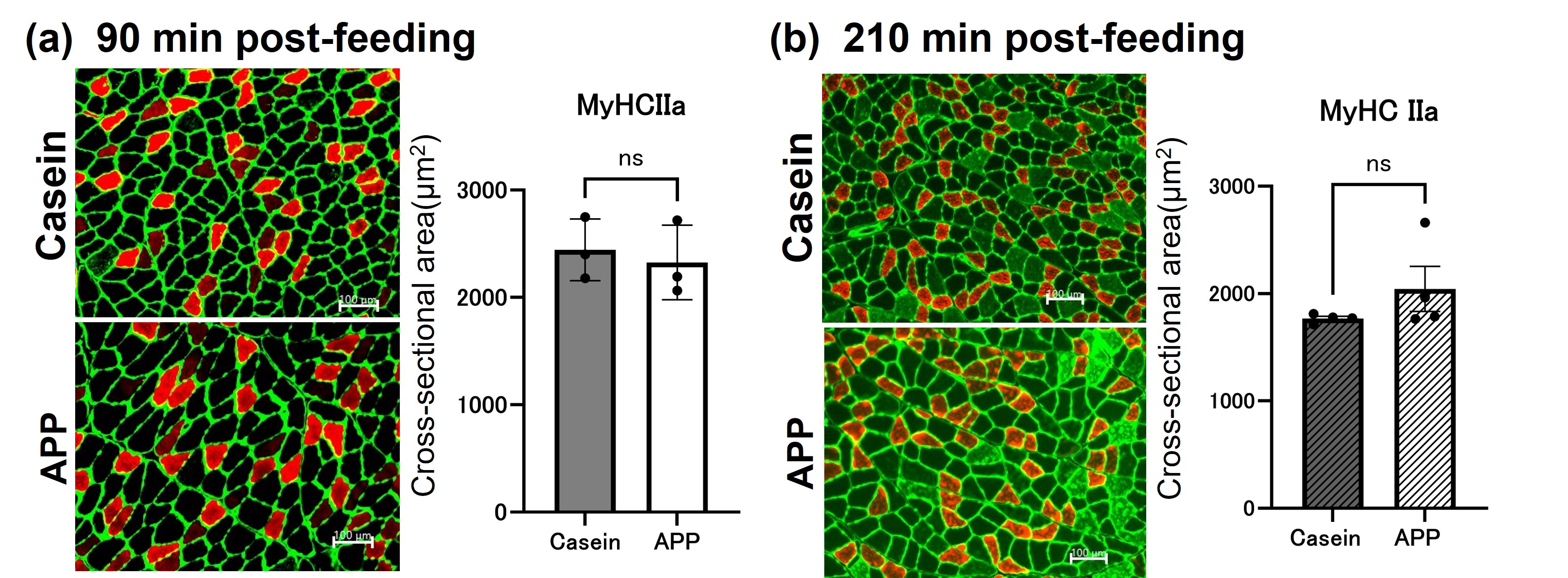

Supplement: S1 Fig — Immunofluorescence staining of the deep region of the gastrocnemius muscle at (a) 90 and (b) 210 min post-feeding. Black indicates MyHC IIx–positive fibers, green indicates MyHC IIb–positive fibers, and red indicates MyHC IIa-positive fibers. The cross-sectional area of red fibers was calculated, and data are presented as the mean ± SEM. Statistical comparisons were performed using Student’s t-test (90 min: N = 3; 210 min: N = 4). *; p < 0.05, **; p < 0.01. Scale bar = 100 μm. (JPG) [file pone.0348366.s001.jpg]

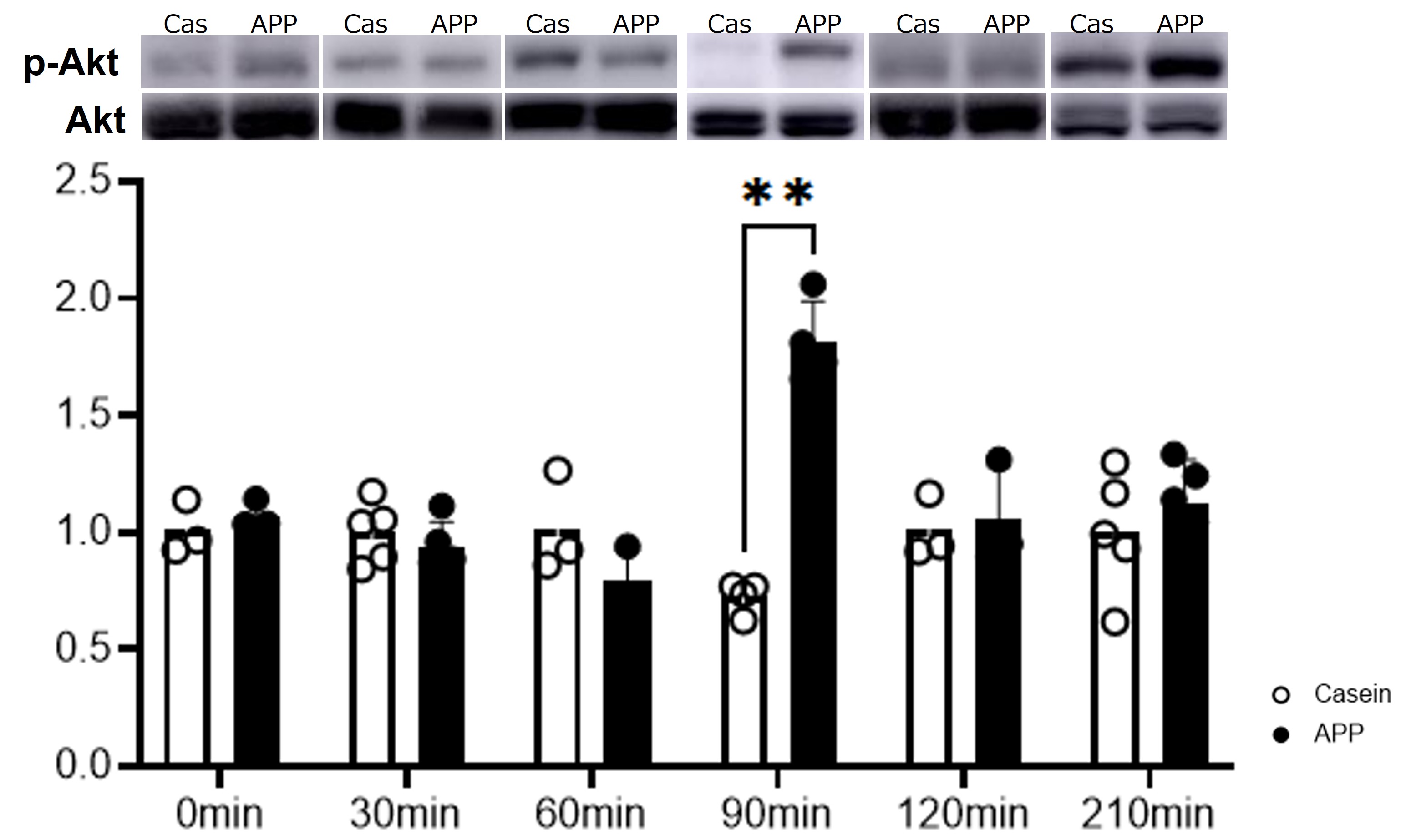

Supplement: S2 Fig — The ratio of phosphorylated Akt to total Akt (pAkt/Akt) in skeletal muscle was measured at 0, 30, 60, 90, 120, and 210 min after feeding. Data are presented as the mean ± SEM. Two-way ANOVA revealed significant effects of time and diet, as well as a significant interaction between time and diet. Post hoc multiple comparisons using Sidak’s test indicated a significant difference between dietary groups at 90 min only. * * P < 0.01. (JPG) [file pone.0348366.s002.jpg]
